# Supplementary figures and images for: Phylogenetic and Comparative Genomics Study of Cephalopina titillator Based on Mitochondrial Genomes
Source: Insects. 2024 Dec 26;16(1):6. doi: 10.3390/insects16010006 (PMC11766325; doi:10.3390/insects16010006)

### Sequencing Depth and Coverage Map

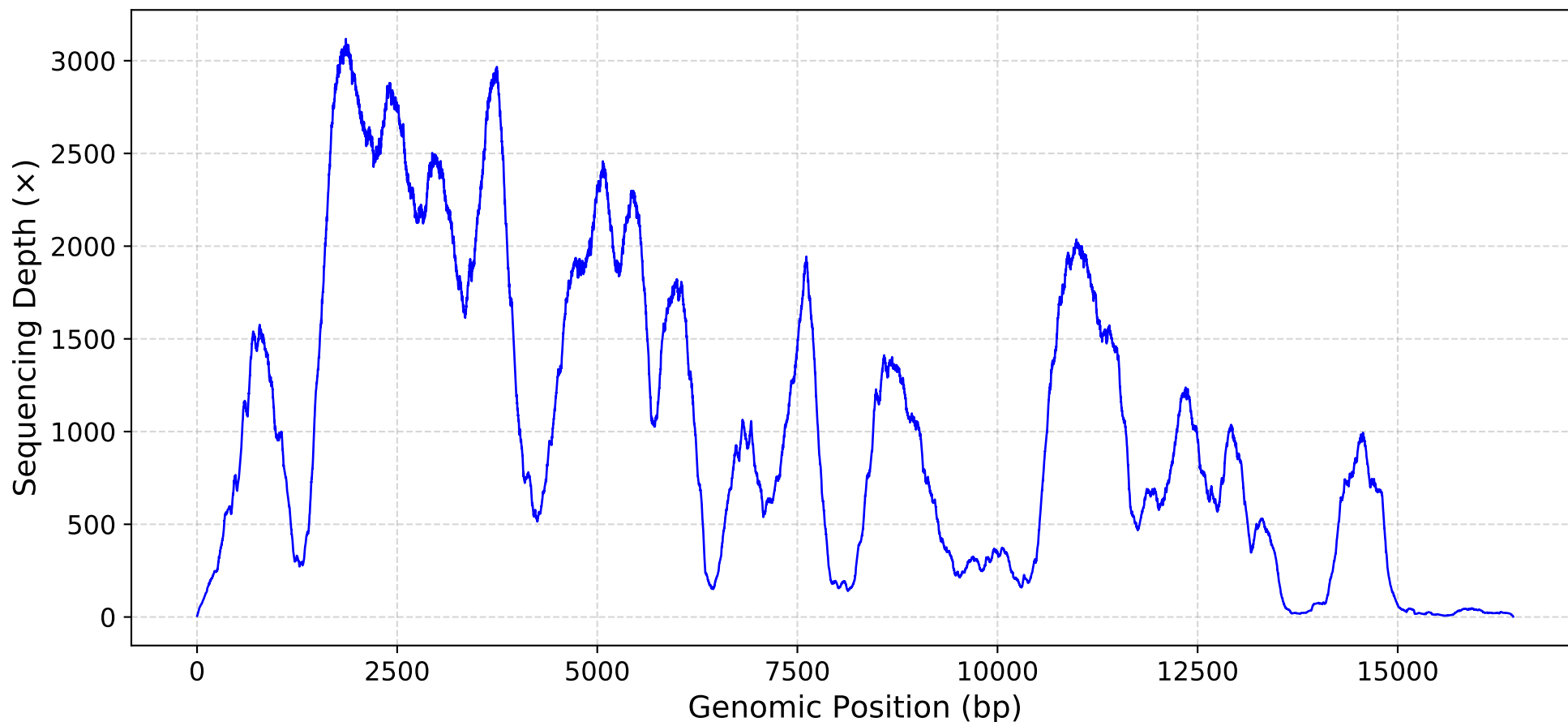

### Sequencing Depth and Coverage Map

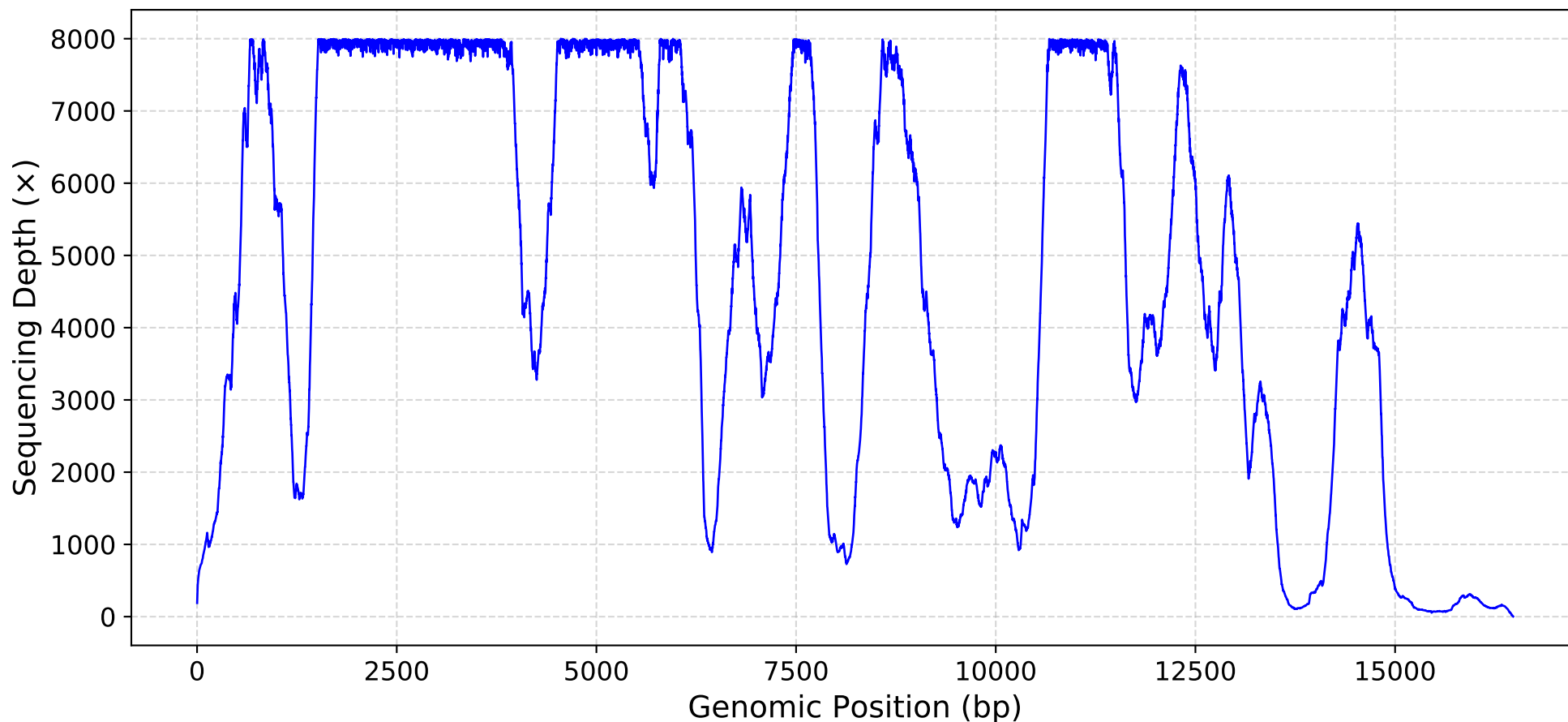

Supplement: Supplementary file 1 [file insects-16-00006-s001.zip › insects-3341487 Supplementary/Figure S1 Sequencing depth and coverage map..pdf]
